# (2-Amino-4,6-dimethyl­pyrimidine-κ*N* ^1^)(2-amino-4-methyl­pyrimidine-κ*N* ^1^)silver(I) perchlorate

**DOI:** 10.1107/S1600536809035193

**Published:** 2009-09-05

**Authors:** Hua Yang

**Affiliations:** a Department of Chemistry, Mudanjiang Teachers College, Mudanjiang 157012, People’s Republic of China

## Abstract

Colourless crystals of the title mixed ligand complex, [Ag(C_5_H_7_N_3_)(C_6_H_9_N_3_)]ClO_4_, were obtained from a solution of 2-amino-4-methyl­pyrimidine, 2-amino-4,6-dimethyl­pyrim­idine and silver perchlorate in water and methanol. The crystal structure is stabilized by inter­molecular N—H⋯O and N—H⋯N hydrogen bonds and π–π stacking inter­actions of the aromatic rings of the two ligands [inter­planar distance = 3.652 (10) Å]. The Ag^I^ atom shows a linear coordination [N—Ag—N = 174.6 (1)°].

## Related literature

For N—Ag—N geometry, see: Greenwood & Earnshaw (1997 ▶). For π–π stacking, see: Munakata *et al.* (2000 ▶). For silver coordination networks, see: Shimizu *et al.* (1999 ▶); Seward *et al.* (2004 ▶).
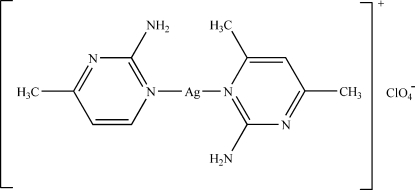


## Experimental

### 

#### Crystal data

[Ag(C_5_H_7_N_3_)(C_6_H_9_N_3_)]ClO_4_
*M* *_r_* = 439.62
Monoclinic, 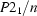

*a* = 12.3952 (5) Å
*b* = 7.8324 (4) Å
*c* = 15.9956 (5) Å
β = 94.339 (3)°
*V* = 1548.47 (11) Å^3^
*Z* = 4
Mo *K*α radiation
μ = 1.50 mm^−1^
*T* = 120 K
0.40 × 0.40 × 0.25 mm

#### Data collection

Bruker APEXII diffractometer
Absorption correction: multi-scan (*SADABS*; Bruker, 2005 ▶) *T*_min_ = 0.553, *T*_max_ = 0.678
8880 measured reflections
2678 independent reflections
2254 reflections with *I* > 2σ(*I*)
*R*_int_ = 0.028

#### Refinement

*R*[*F* ^2^ > 2σ(*F* ^2^)] = 0.037
*wR*(*F* ^2^) = 0.100
*S* = 1.07
2678 reflections
211 parameters
H-atom parameters constrained
Δρ_max_ = 1.36 e Å^−3^
Δρ_min_ = −0.53 e Å^−3^

### 

Data collection: *APEX2* (Bruker, 2005 ▶); cell refinement: *SAINT* (Bruker, 2005 ▶); data reduction: *SAINT*; program(s) used to solve structure: *SHELXS97* (Sheldrick, 2008 ▶); program(s) used to refine structure: *SHELXL97* (Sheldrick, 2008 ▶); molecular graphics: *ORTEP-3 for Windows* (Farrugia, 1997 ▶); software used to prepare material for publication: *WinGX* (Farrugia, 1999 ▶).

## Supplementary Material

Crystal structure: contains datablocks I, global. DOI: 10.1107/S1600536809035193/ng2621sup1.cif

Structure factors: contains datablocks I. DOI: 10.1107/S1600536809035193/ng2621Isup2.hkl

Additional supplementary materials:  crystallographic information; 3D view; checkCIF report

## Figures and Tables

**Table 1:** Hydrogen-bond geometry (Å, °)

*D*—H⋯*A*	*D*—H	H⋯*A*	*D*⋯*A*	*D*—H⋯*A*
N5—H5*C*⋯O4^i^	0.86	2.32	3.131 (5)	158
N5—H5*B*⋯N3^ii^	0.86	2.20	3.050 (5)	172
N2—H2*B*⋯O2	0.86	2.50	3.077 (5)	126
N2—H2*A*⋯N6^iii^	0.86	2.30	3.147 (5)	169

Symmetry codes: (i) 
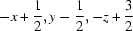
; (ii) 
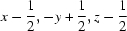
; (iii) 
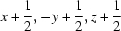
.

**Fig. 1.**
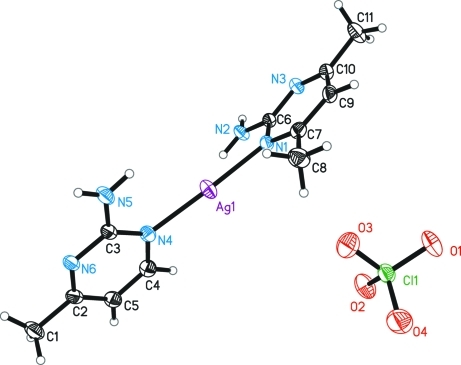
The molecular structure with atom labels and 30% probability displacement ellipsoids for non-H atoms.

**Fig. 2.**
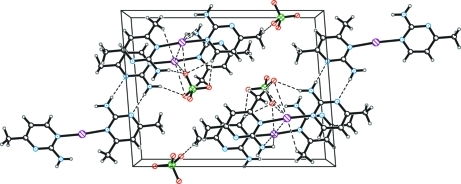
The packing diagram of molecules, viewed down the b axis, with the weak interactions shown as dashed lines.

## supplementary crystallographic information

### Comment

The structure of the title compound (I) comprises of uncoordinated ClO_4_^-^ anions and [Ag(2-amino-4-methylpyrimidine)(2-amino-4,6-dimethylpyrimidine)]^+^ cations. The central silver(I) ion, possessing its vacant s and p orbitals, coordinated to two nitrogen atoms from those two different pyrimidine derivative ligands, presenting nearly linear N-Ag-N geometry Greenwood *et al.*, 1997). An one dimensional framework was built by multiple intermolecular N–H–N hydrogen bonds along one of the diagonals of a and c axial plane, while pi–pi stacking interaction of the aromatic rings with an interplane distance 3.65 Å stabilized the whole crystal structure (Munakata *et al.*, 2000).

### Experimental

A solution of 108 mg (1 mmol) 2-amino-4-methylpyrimidine and 123 mg (1 mmol) of 2-amino-4,6-dimethylpyrimidine in distilled water-CH_3_OH (1:1 v/v, 10 mL) was added to an aqueous solution of AgClO_4_ 208 mg (1 mmol) in 3 ml distilled water at 333 K. A small amount of white precipitate was removed from the resulting solution. Prism colorless crystals were obtained by slow evaporation at room temperature over a period of 3 days.

### Refinement

All H atoms were placed in calculated positions and refined as riding, with C–H = 0.96–0.98 Å, and N–H = 0.86 Å, and *U*_iso_(H) = 1.2 or 1.5*U*_eq_(C,*N*). The final difference map had a peak near Ag1.

### Crystal data

**Table:**

[Ag(C_5_H_7_N_3_)(C_6_H_9_N_3_)]ClO_4_	*F*(000) = 880
*M**_r_* = 439.62	*D*_x_ = 1.886 Mg m^−^^3^
Monoclinic, *P*2_1_/*n*	Mo *K*α radiation, λ = 0.71073 Å
Hall symbol: -P 2yn	Cell parameters from 2795 reflections
*a* = 12.3952 (5) Å	θ = 2.6–32.8°
*b* = 7.8324 (4) Å	µ = 1.50 mm^−^^1^
*c* = 15.9956 (5) Å	*T* = 120 K
β = 94.339 (3)°	Prism, colourless
*V* = 1548.47 (11) Å^3^	0.40 × 0.40 × 0.25 mm
*Z* = 4	

### Data collection

**Table:**

Bruker APEXII diffractometer	2678 independent reflections
Radiation source: fine-focus sealed tube	2254 reflections with *I* > 2σ(*I*)
graphite	*R*_int_ = 0.028
φ and ω scans	θ_max_ = 25.0°, θ_min_ = 2.6°
Absorption correction: multi-scan (*SADABS*; Bruker, 2005)	*h* = −14→14
*T*_min_ = 0.553, *T*_max_ = 0.678	*k* = −8→9
8880 measured reflections	*l* = −19→19

### Refinement

**Table:**

Refinement on *F*^2^	Primary atom site location: structure-invariant direct methods
Least-squares matrix: full	Secondary atom site location: difference Fourier map
*R*[*F*^2^ > 2σ(*F*^2^)] = 0.037	Hydrogen site location: inferred from neighbouring sites
*wR*(*F*^2^) = 0.100	H-atom parameters constrained
*S* = 1.07	*w* = 1/[σ^2^(*F*_o_^2^) + (0.0544*P*)^2^ + 2.3112*P*] where *P* = (*F*_o_^2^ + 2*F*_c_^2^)/3
2678 reflections	(Δ/σ)_max_ = 0.001
211 parameters	Δρ_max_ = 1.36 e Å^−^^3^
0 restraints	Δρ_min_ = −0.52 e Å^−^^3^

### Special details

**Table:**

Geometry. All e.s.d.'s (except the e.s.d. in the dihedral angle between two l.s. planes) are estimated using the full covariance matrix. The cell e.s.d.'s are taken into account individually in the estimation of e.s.d.'s in distances, angles and torsion angles; correlations between e.s.d.'s in cell parameters are only used when they are defined by crystal symmetry. An approximate (isotropic) treatment of cell e.s.d.'s is used for estimating e.s.d.'s involving l.s. planes.
Refinement. Refinement of *F*^2^ against ALL reflections. The weighted *R*-factor *wR* and goodness of fit *S* are based on *F*^2^, conventional *R*-factors *R* are based on *F*, with *F* set to zero for negative *F*^2^. The threshold expression of *F*^2^ > σ(*F*^2^) is used only for calculating *R*-factors(gt) *etc*. and is not relevant to the choice of reflections for refinement. *R*-factors based on *F*^2^ are statistically about twice as large as those based on *F*, and *R*- factors based on ALL data will be even larger.

### Fractional atomic coordinates and isotropic or equivalent isotropic displacement parameters (Å^2^)

**Table:**

	*x*	*y*	*z*	*U*_iso_*/*U*_eq_	
Ag1	0.19079 (3)	0.45483 (5)	0.720510 (19)	0.04423 (15)	
C1	0.1554 (4)	0.3648 (7)	0.3245 (3)	0.0544 (12)	
H1A	0.1108	0.2682	0.3087	0.082*	
H1B	0.2261	0.3487	0.3051	0.082*	
H1C	0.1235	0.4663	0.2998	0.082*	
C2	0.1644 (3)	0.3818 (5)	0.4169 (3)	0.0373 (9)	
C3	0.0929 (3)	0.3330 (5)	0.5423 (2)	0.0332 (9)	
C4	0.2530 (4)	0.4782 (6)	0.5433 (3)	0.0398 (10)	
H4A	0.3106	0.5345	0.5720	0.048*	
C5	0.2516 (4)	0.4648 (6)	0.4585 (3)	0.0410 (10)	
H5A	0.3073	0.5097	0.4294	0.049*	
C6	0.3056 (3)	0.4035 (5)	0.8973 (2)	0.0316 (8)	
C7	0.1481 (3)	0.5552 (5)	0.9020 (3)	0.0369 (9)	
C8	0.0587 (4)	0.6489 (7)	0.8581 (3)	0.0566 (13)	
H8A	0.0871	0.7365	0.8240	0.085*	
H8B	0.0149	0.7000	0.8983	0.085*	
H8C	0.0153	0.5717	0.8231	0.085*	
C9	0.1577 (4)	0.5429 (6)	0.9877 (3)	0.0419 (10)	
H9A	0.1075	0.5953	1.0197	0.050*	
C10	0.2424 (4)	0.4521 (5)	1.0253 (3)	0.0380 (9)	
C11	0.2548 (5)	0.4273 (8)	1.1177 (3)	0.0606 (14)	
H11A	0.3269	0.4582	1.1384	0.091*	
H11B	0.2420	0.3097	1.1307	0.091*	
H11C	0.2037	0.4979	1.1437	0.091*	
N1	0.2212 (3)	0.4824 (4)	0.8553 (2)	0.0319 (7)	
N2	0.3831 (3)	0.3336 (4)	0.8522 (2)	0.0383 (8)	
H2A	0.4366	0.2798	0.8774	0.046*	
H2B	0.3785	0.3430	0.7985	0.046*	
N3	0.3176 (3)	0.3841 (5)	0.9803 (2)	0.0365 (8)	
N4	0.1743 (3)	0.4133 (4)	0.5875 (2)	0.0350 (8)	
N5	0.0136 (3)	0.2635 (5)	0.5824 (2)	0.0455 (9)	
H5B	−0.0386	0.2113	0.5545	0.055*	
H5C	0.0144	0.2708	0.6360	0.055*	
N6	0.0857 (3)	0.3161 (4)	0.4580 (2)	0.0361 (8)	
Cl1	0.50494 (9)	0.64276 (14)	0.68793 (6)	0.0412 (3)	
O1	0.4928 (3)	0.6816 (5)	0.6012 (2)	0.0629 (10)	
O2	0.4001 (3)	0.6130 (5)	0.7187 (2)	0.0617 (9)	
O3	0.5697 (3)	0.4961 (5)	0.7031 (3)	0.0666 (10)	
O4	0.5523 (4)	0.7822 (6)	0.7327 (3)	0.0775 (12)	

### Atomic displacement parameters (Å^2^)

**Table:**

	*U*^11^	*U*^22^	*U*^33^	*U*^12^	*U*^13^	*U*^23^
Ag1	0.0420 (2)	0.0623 (3)	0.02780 (19)	−0.00167 (16)	−0.00078 (13)	−0.00457 (14)
C1	0.062 (3)	0.071 (3)	0.030 (2)	−0.007 (3)	0.003 (2)	0.006 (2)
C2	0.044 (2)	0.037 (2)	0.031 (2)	0.0077 (19)	0.0058 (18)	0.0064 (17)
C3	0.036 (2)	0.035 (2)	0.0282 (19)	0.0065 (17)	0.0038 (16)	0.0023 (16)
C4	0.035 (2)	0.043 (2)	0.041 (2)	0.0017 (19)	−0.0023 (18)	0.0003 (19)
C5	0.041 (2)	0.045 (2)	0.038 (2)	−0.001 (2)	0.0064 (19)	0.0027 (19)
C6	0.036 (2)	0.032 (2)	0.0266 (19)	−0.0052 (17)	0.0030 (16)	−0.0023 (15)
C7	0.038 (2)	0.031 (2)	0.041 (2)	−0.0037 (18)	0.0006 (18)	−0.0006 (17)
C8	0.060 (3)	0.062 (3)	0.047 (3)	0.008 (3)	0.001 (2)	0.007 (2)
C9	0.049 (3)	0.038 (2)	0.041 (2)	−0.005 (2)	0.018 (2)	−0.0107 (19)
C10	0.045 (2)	0.037 (2)	0.032 (2)	−0.004 (2)	0.0037 (18)	−0.0054 (17)
C11	0.074 (4)	0.078 (4)	0.031 (2)	0.008 (3)	0.009 (2)	−0.006 (2)
N1	0.0344 (17)	0.0330 (17)	0.0285 (16)	−0.0038 (14)	0.0044 (14)	−0.0037 (13)
N2	0.0432 (19)	0.047 (2)	0.0250 (16)	0.0066 (16)	0.0074 (14)	0.0008 (14)
N3	0.0381 (18)	0.0430 (19)	0.0288 (17)	−0.0001 (16)	0.0050 (14)	−0.0035 (15)
N4	0.0326 (17)	0.0402 (19)	0.0318 (17)	0.0028 (15)	−0.0010 (14)	0.0000 (14)
N5	0.047 (2)	0.063 (2)	0.0269 (17)	−0.0130 (19)	0.0049 (15)	−0.0005 (17)
N6	0.0416 (19)	0.0403 (19)	0.0264 (16)	0.0019 (16)	0.0011 (14)	0.0031 (14)
Cl1	0.0467 (6)	0.0444 (6)	0.0331 (5)	−0.0052 (5)	0.0068 (4)	0.0038 (4)
O1	0.064 (2)	0.090 (3)	0.0356 (17)	−0.004 (2)	0.0119 (16)	0.0148 (17)
O2	0.053 (2)	0.086 (3)	0.049 (2)	−0.0050 (19)	0.0193 (16)	0.0121 (18)
O3	0.067 (2)	0.058 (2)	0.074 (3)	0.0125 (18)	0.000 (2)	0.0080 (19)
O4	0.092 (3)	0.077 (3)	0.063 (2)	−0.031 (2)	−0.001 (2)	−0.007 (2)

### Geometric parameters (Å, °)

**Table:**

Ag1—N4	2.146 (3)	C7—C8	1.465 (7)
Ag1—N1	2.171 (3)	C8—H8A	0.9600
C1—C2	1.479 (6)	C8—H8B	0.9600
C1—H1A	0.9600	C8—H8C	0.9600
C1—H1B	0.9600	C9—C10	1.370 (7)
C1—H1C	0.9600	C9—H9A	0.9300
C2—N6	1.322 (6)	C10—N3	1.330 (6)
C2—C5	1.387 (6)	C10—C11	1.487 (6)
C3—N5	1.330 (6)	C11—H11A	0.9600
C3—N6	1.351 (5)	C11—H11B	0.9600
C3—N4	1.352 (5)	C11—H11C	0.9600
C4—N4	1.347 (6)	N2—H2A	0.8600
C4—C5	1.360 (6)	N2—H2B	0.8600
C4—H4A	0.9300	N5—H5B	0.8600
C5—H5A	0.9300	N5—H5C	0.8600
C6—N3	1.334 (5)	Cl1—O4	1.409 (4)
C6—N1	1.350 (5)	Cl1—O3	1.412 (4)
C6—N2	1.359 (5)	Cl1—O1	1.417 (3)
C7—N1	1.344 (6)	Cl1—O2	1.443 (4)
C7—C9	1.370 (6)		
			
N4—Ag1—N1	174.61 (13)	C7—C9—H9A	120.6
C2—C1—H1A	109.5	C10—C9—H9A	120.6
C2—C1—H1B	109.5	N3—C10—C9	121.0 (4)
H1A—C1—H1B	109.5	N3—C10—C11	117.4 (4)
C2—C1—H1C	109.5	C9—C10—C11	121.5 (4)
H1A—C1—H1C	109.5	C10—C11—H11A	109.5
H1B—C1—H1C	109.5	C10—C11—H11B	109.5
N6—C2—C5	121.4 (4)	H11A—C11—H11B	109.5
N6—C2—C1	117.3 (4)	C10—C11—H11C	109.5
C5—C2—C1	121.3 (4)	H11A—C11—H11C	109.5
N5—C3—N6	116.4 (4)	H11B—C11—H11C	109.5
N5—C3—N4	118.8 (4)	C7—N1—C6	116.6 (3)
N6—C3—N4	124.7 (4)	C7—N1—Ag1	121.4 (3)
N4—C4—C5	122.7 (4)	C6—N1—Ag1	121.2 (3)
N4—C4—H4A	118.6	C6—N2—H2A	120.0
C5—C4—H4A	118.6	C6—N2—H2B	120.0
C4—C5—C2	117.7 (4)	H2A—N2—H2B	120.0
C4—C5—H5A	121.1	C10—N3—C6	117.6 (4)
C2—C5—H5A	121.1	C4—N4—C3	115.8 (4)
N3—C6—N1	124.8 (4)	C4—N4—Ag1	116.4 (3)
N3—C6—N2	116.9 (4)	C3—N4—Ag1	127.7 (3)
N1—C6—N2	118.2 (3)	C3—N5—H5B	120.0
N1—C7—C9	121.1 (4)	C3—N5—H5C	120.0
N1—C7—C8	117.6 (4)	H5B—N5—H5C	120.0
C9—C7—C8	121.3 (4)	C2—N6—C3	117.6 (4)
C7—C8—H8A	109.5	O4—Cl1—O3	109.5 (3)
C7—C8—H8B	109.5	O4—Cl1—O1	109.9 (2)
H8A—C8—H8B	109.5	O3—Cl1—O1	111.1 (3)
C7—C8—H8C	109.5	O4—Cl1—O2	107.6 (3)
H8A—C8—H8C	109.5	O3—Cl1—O2	109.0 (3)
H8B—C8—H8C	109.5	O1—Cl1—O2	109.6 (2)
C7—C9—C10	118.8 (4)		

### Hydrogen-bond geometry (Å, °)

**Table:**

*D*—H···*A*	*D*—H	H···*A*	*D*···*A*	*D*—H···*A*
N5—H5C···O4^i^	0.86	2.32	3.131 (5)	158
N5—H5B···N3^ii^	0.86	2.20	3.050 (5)	172
N2—H2B···O2	0.86	2.50	3.077 (5)	126
N2—H2A···N6^iii^	0.86	2.30	3.147 (5)	169
C1—H1C···O4^iv^	0.96	2.38	3.339 (7)	177
C4—H4A···O1	0.93	2.55	3.437 (6)	160
C4—H4A···O2	0.93	2.59	3.396 (6)	145
C8—H8C···O4^i^	0.96	2.55	3.456 (7)	157

Symmetry codes: (i) −*x*+1/2, *y*−1/2, −*z*+3/2; (ii) *x*−1/2, −*y*+1/2, *z*−1/2; (iii) *x*+1/2, −*y*+1/2, *z*+1/2; (iv) *x*−1/2, −*y*+3/2, *z*−1/2.
